# Characterization of the Complete Chloroplast Genome Sequences of Four *Zanthoxylum* L. Species (Sapindales: Rutaceae) from the Caribbean, Madagascar, the Mascarene Islands, and the South Pacific

**DOI:** 10.1128/MRA.00399-21

**Published:** 2021-05-27

**Authors:** Niklas Reichelt, Jun Wen, Claudia Pätzold, Marc S. Appelhans

**Affiliations:** a Department of Systematics, Biodiversity and Evolution of Plants, Albrecht-von-Haller-Institute of Plant Sciences, University of Goettingen, Goettingen, Germany; b Department of Botany, National Museum of Natural History, Smithsonian Institution, Washington, DC, USA; c Department of Botany and Molecular Evolution, Senckenberg Research Institute and Natural History Museum Frankfurt, Frankfurt am Main, Germany; Vanderbilt University

## Abstract

*Zanthoxylum* is a genus of woody plants in the Rutaceae family distributed pantropically, with some species extending to temperate regions in East Asia and North America. Here, we present the complete chloroplast genome sequences of four species, two of them critically endangered, endemic to tropical islands.

## ANNOUNCEMENT

*Zanthoxylum* L. (prickly ash, Sichuan pepper) is the second largest genus within the Rutaceae and is distributed pantropically, with some species in temperate Asia and North America (1). The genus occurs on a variety of island systems, including, e.g., the remote Hawaiian, Austral, and Juan Fernández Islands. This study provides genetic resources for the following four *Zanthoxylum* species: Z. tragodes, from the Caribbean and Venezuela (2); Z. madagascariense, endemic to Madagascar (3); Z. pinnatum, from the South Pacific, with the category critically endangered (CR) proposed for the Austral Island population (4); and Z. paniculatum, endemic to Rodrigues Island (Mauritius), with just a few specimens known today (5). Applying the International Union for Conservation of Nature (IUCN) red list criteria (6), *Z. paniculatum* should be considered CR.

Total DNA was extracted from 1 cm^2^ herbarium leaf material from *Z. madagascariense* (collector and number, Capuron 28595-SF; country of origin, Madagascar; year of collection, 1968; herbarium, MO [herbarium codes are assigned according to the Index Herbariorum, http://sweetgum.nybg.org/science/ih]), *Z. paniculatum* (collector and number, C. Magdalena 001; country of origin, Mauritius [Rodrigues]; year of collection, 2007; herbarium, MO), *Z. pinnatum* (collector and number, Drake 282; country of origin, Tonga [Vava’u group]; year of collection, 1995; herbarium, US), and *Z. tragodes* (collector and number, Liogier 12644; country of origin, Dominican Republic; year of collection, 1968; herbarium, US) using the cetyltrimethylammonium bromide (CTAB) method (7). The NEBNext Ultra II DNA library prep kit for Illumina (New England Biolabs, USA) and NEBNext multiplex oligos for Illumina (dual-index primers set 1) were employed for size selection and adapter index ligation. Bait and library hybridization were performed according to the myBaits Hybridization Capture for Targeted NGS manual v.4.01 (Arbor Biosciences, USA), using a bait set designed for nuclear genes of *Zanthoxylum* and related genera (8). Plastome genome data gathered in this process were off-target hits. The captured DNA libraries were amplified with the KAPA HiFi HotStart ReadyMix (Hoffmann-La Roche, Switzerland). Target-enriched libraries were sequenced on a HiSeq 4000 instrument (Illumina, Inc., USA), producing 2 × 150-bp paired-end reads.

Trimmomatic v.0.33 (9) was used for adapter and quality trimming (PHRED33 score, >30; minimum length after trimming, 65 bp). FastUniq v.1.1 (10) was employed for deduplication. Using Fast-Plast v.1.2.8 (11), reads were mapped to all Sapindales plastomes available in GenBank with a required minimum coverage of 5×. Annotations were drawn from the Zanthoxylum bungeanum (GenBank accession number KX497031) and *Phellodendron amurense* (NC_035551) references in Geneious v.10.0.8 using the “live annotate and predict” function, followed by manual annotation of unrecognized regions. The chloroplast genomes were visualized using OrganellarGenomeDRAW (12).

Sequencing produced between 9,939,558 and 15,323,260 reads per sample after quality trimming. In all, 25,098 to 79,647 reads mapped to the *Z. bungeanum* reference genome. This corresponds to a plastome coverage of about 47× to 151×. All plastomes show the typical structure with a large single-copy (LSC) and small single-copy (SSC) region separated by two inverted repeats (IRA and IRB). Plastome lengths range from 158,265 bp in *Z. paniculatum* to 158,722 bp in Z. *pinnatum*. The total GC contents are consistent for all four plastomes at 38.4%. In each plastome, a total of 132 genes were annotated, 40 of which are situated in the IRA and IRB regions. Genes in the two inverted repeat regions comprise all 8 rRNAs, 14 of 37 tRNAs, and 20 of 87 protein-coding genes. The gene *ycf1* within the IRA differs from its copy within the IRB, as it deeply extends into the SSC region. The protein-coding gene *ndhF* is partly situated within an inverted repeat. The protein-coding gene *rps12* is trans-spliced, and its two transcripts are found in the LSC and IR regions.

### Data availability.

The chloroplast genome sequence data were deposited in GenBank (*Z. madagascariense*, accession number MN968551; *Z. paniculatum*, MN968552; *Z. pinnatum*, MN968553; *Z. tragodes*, MN968554). The demultiplexed raw data were deposited at the NCBI Sequence Read Archive (BioProject accession number PRJNA623909).
